# Making the Best of a Pest: The Potential for Using Invasive Zebra Mussel (*Dreissena Polymorpha)* Biomass as a Supplement to Commercial Chicken Feed

**DOI:** 10.1007/s00267-014-0335-6

**Published:** 2014-08-09

**Authors:** Claire McLaughlan, Paul Rose, David C. Aldridge

**Affiliations:** 1 Department of Zoology, University of Cambridge, Downing Street, Cambridge, CB2 3EJ UK; 2 Department of Animal Production, Welfare and Veterinary Sciences, Harper Adams University College, Newport, Shropshire, TF10 8NB UK

**Keywords:** *Dreissena polymorpha*, Poultry feed, Calcium, Biomanipulation, Invasive species

## Abstract

Invasive non-native species frequently occur in very high densities. When such invaders present an economic or ecological nuisance, this biomass is typically removed and landfill is the most common destination, which is undesirable from both an economic and ecological perspective. The zebra mussel, *Dreissena polymorpha*, has invaded large parts of Europe and North America, and is routinely removed from raw water systems where it creates a biofouling nuisance. We investigated the suitability of dried, whole zebra mussels as a supplement to poultry feed, thus providing a more attractive end-use than disposal to landfill. Measurable outcomes were nutrient and energy composition analyses of the feeds and production parameters of the birds over a 14 day period. Zebra mussels were a palatable feed supplement for chickens. The mussel meal contained high levels of calcium (344.9 g kg^−1^), essential for egg shell formation, which was absorbed and retained easily by the birds. Compared with standard feed, a mussel-supplemented diet caused no significant effects on production parameters such as egg weight and feed conversion ratio during the study period. However, protein and energy levels in the zebra mussel feed were much lower than expected from the literature. In order for zebra mussels to be a viable long-term feed supplement for poultry, flesh would need to be separated from the shells in an economically viable way. If zebra mussels were to be used with the shells remaining, it seems that the resultant mussel meal would be more suitable as a calcium supplement.

## Introduction

Invasive non-native species characteristically comprise a high proportion of the biomass in the systems they inhabit. This is particularly true in aquatic habitats. Bivalves, for example, are notorious biofoulers; the Asian clam, *Corbicula fluminea,* has been observed at mean annual biomasses of 160 g DW m^−2^ in the River Minho, Portugal (Sousa et al. 2008). The Asian date mussel (*Musculista senhousia*) typically forms mats on the seabed with densities of 5,000–10,000 ind m^−2^ (Crooks 2002). When biofouling becomes a problem, either in terms of compromised ecosystem functioning or for economic reasons, physical removal of the organism is often the main control method, and the biomass must then be safely disposed of. Landfill is typically the default disposal option, however, there is growing awareness that removed biomass of invasive species may be utilised for other more beneficial purposes. For example, in developing countries, water hyacinth can be a low cost and nutritious source of fodder for pigs (Men et al. 2002) and goats (Dada 2002). Other invasive species, such as the crayfish *Procambarus clarkii*, are harvested for food (Geiger et al. 2005).

The zebra mussel, *Dreissena polymorpha*, is widely recognised as one of the most prolific freshwater invasive species. Native to the Ponto-Caspian region of Eastern Europe, it is now widely established within North America and Western Europe (Gallardo et al. 2013) where it continues to spread (e.g., Aldridge et al. 2006). The species can occur in densities as high as 750,000 individuals m^−2^ (Kovalak et al. 1993), driving ecosystem-level change (Sousa et al. 2009) and becoming a costly biofouler to industry (Elliott et al. 2005; Strayer 2009). Reactive methods of control for biofouling zebra mussels include mechanical removal, high-pressure water cleaning (Mackie and Claudie 2010) and microencapsulated BioBullets (Aldridge et al. 2006), with the removed mussels typically disposed of as non-hazardous landfill. An additional emerging source of zebra mussel biomass comes from schemes where zebra mussels are cultivated to use their capabilities as filter feeders to reduce eutrophication (Stybel et al. 2009; McLaughlan and Aldridge 2013). Such schemes include harvesting the mussels to permanently remove nutrients from the water, therefore providing another large source of mussel material. Given the large biomass of zebra mussels removed by industry (in 2008 one UK waterworks took 778 tonnes from a 4.5 km pipe transporting water to the works), the cost of landfill (£80 per tonne in the UK, at 2014 prices; HMRC, 2013), and the rising environmental sensitivities to such disposal, there is a desire to find alternative end uses.

One possible use for zebra mussel biomass is as a component of poultry feed. Chickens require a constant intake of high-quality protein for growth and productivity. Conventional diets are largely cereal-based, supplemented with protein components such as soya, wheat, and fishmeal. Egg-laying hens also require diets enriched with calcium to assist shell production. There is reason to believe that mussels may provide a viable alternative source of protein and calcium; the flesh of blue mussels, *Mytilus edulis*, has been shown to have a similar amino acid profile to fishmeal (Jonsson and Elwinger 2009), and the dried flesh has been used in poultry feed trials with very promising results (Jönsson et al. 2011). The literature suggests that zebra mussels are also high in protein (Secor et al. 1993), and therefore it is reasonable to believe they would make a suitable feedstuff.

The aim of this study was therefore to determine whether zebra mussels could represent a suitable feedstuff to make up a component of the diet of laying poultry. Such an end-use would offer considerable economic and sustainability benefit over disposal to landfill. Protein, energy, and nutrient levels in the mussels were determined, and the feed was given to laying hens for a trial period. The palatability, digestibility, and effects on production of the feed were compared against a conventional diet. Whole mussels (flesh and shell) were trialled in this study.

## Materials and Methods

### Mussel Collection and Feed Preparation

Zebra mussels were collected in September 2012 from a bridge in the village of Cattawade on the River Stour in Suffolk, UK (NGR: TM1017733041), and supplemented with material from the River Thames, UK (NGR: TQ1782073556). All specimens were killed by freezing. Due to limited availability of mussels, those used did vary in size from approx. 2040 mm (they were all adults). It is acknowledged that size and age could affect the proportion of flesh to shell. Defrosted mussels were rinsed and the shells cleaned, before being dried to constant mass at 60 °C for 24 h. The whole-dried mussels were ground to a fine powder using a lab mill with a 2 mm mesh diameter. This process yielded 4.8 kg of mussel meal. It should be noted that higher temperatures (80–85 °C) may be required for approval in commercial feed production, in order to ensure that all bacteria are killed.

Feeds (control, 7.5 and 15 % mussel meal) were formulated. The control feed was a commercial, low protein, wheat-based feed (Layers mash, code 116: Target Feeds Ltd., Whitchurch, Shropshire, UK). The components of this feed were wheat (68 %), soya (20 %), soya oil (1 %), calcium phosphate (1 %), limestone (8.5 %), salt (0.3 %), lysine (0.1 %, methionine (0.2 %), and threonine (0.1 %). To create the two experimental feeds, 7.5 and 15 % mussel meal was added to the standard feed, respectively; directly substituted for the same amount of standard feed. Therefore, the composition of the feed will have been altered, however, our aim was to look for any deleterious or positive effects of adding mussel meal to the diet and to see how well it was retained by the birds, and this experimental design was sufficient to fulfil this aim. Each feed was mixed for 2 mins to achieve homogeneity.

### Feeding Experiment

Thirty Hy-Line brown laying hens (supplied by Country Fresh Pullets Ltd., Shropshire, UK) were used in the study. They were supplied at 16 weeks of age and raised on a proprietary feed (Target Feeds Ltd., Shropshire, UK) until aged 22 weeks and in lay. They were then fed the control feed for 2 weeks until the start of the trial to allow acclimation. Before the trial commenced, each bird was weighed and placed in a cage assigned using a randomised block design. For the first 10 days of the trial, the appropriate diet (10 birds for each of the three diets) was given. Food and water were freely available at all times. The number of eggs laid per day was recorded, and all other conditions kept constant (temperature 20 °C, 14^L^: 10D light cycle).

One individual, which had been assigned the 15 % mussel meal diet, stopped laying on day three of the trial (the habituation period), and had been eating less food than other birds. Therefore, this bird was removed from the trial before the experimental period began. All other birds were eating and laying satisfactorily.

Days 11–14 of the trial comprised the ‘experimental’ period, where droppings were collected from trays beneath each bird daily, and refrigerated at 4 °C. All eggs were collected on days 11 and 12 and stored. For each bird, the mass of food eaten between days 11 and 14 was measured, and the weight change between days 11 and 14 calculated.

### Lab Analysis

Droppings from each bird over the 4 day experimental period were dried to constant mass at 60 °C for 48 h and ground in a lab mill to a fine powder. Egg quality tests were performed on eggs laid on days 11 and 12. These consisted of egg mass, shell deformation using a Marius instrument (technique developed by Schoorl and Boersma 1962), albumen height using a tripod micrometer (Brant et al. 1951), and yolk color score (using the DSM Yolk Color Fan that was previously called Roche Yolk Color Fan).

Pure mussel meal, basal feed, and dried droppings from each bird were analyzed for gross energy, nitrogen, phosphorus, calcium, oil A (lipids), ash content, and amino acid composition. Calcium was determined by atomic absorption, phosphorus by the UV molybdovanadate method, and nitrogen by the Dumas technique using a Leco combustion instrument. These analyses were carried out by DM Scientific Ltd. (Thirsk, UK). Amino acids were analyzed in the University of Cambridge, Biochemistry Department by a standard method using an ion-exchange analyzer (Biochrom 30). Amino acid composition analysis of the feeds/droppings was able to identify the mass of 16 amino acids present (not including cysteine and tryptophan). Gross energy was determined by combustion. Due to the difficulty of combusting the pure mussel meal sample, a sucrose lab standard was used and zebra mussel meal added at three levels (33, 50, and 75 %).

### Statistical Analysis

The experimental data were compared by diet using one-way analysis of variance for production parameters: egg weight, number of eggs, feed intake, feed conversion, excreta dry mass, excreta % dry mass, and change in bird bodyweight. In the poultry feed industry, it is not only the nutrient and energy content of a feed which must be known, but also the amount of energy which will be metabolized per unit of food intake, and the digestibility of amino acids and levels of macronutrients which will be retained for utilisation by the bird. Apparent metabolizable energy (AME) was therefore calculated by difference, using the amount of energy ingested in the food, the amount excreted in feces, and the amount of food eaten. The same principle was applied to nitrogen retention, phosphorus retention, and calcium retention. Amino acid digestibility was calculated as a coefficient using the intake of the individual amino acid minus the output in droppings, divided by input. The results of these calculations were subjected to one-way analysis of variance to determine differences between diets and subsequent post hoc pairwise comparisons (Bonferroni tests to control for Type I errors with multiple comparisons). Data were shown to approximate to normal distributions (Anderson–Darling *P* > 0.05) and so parametric tests were used. Levene’s test was used to test homogeneity of variances. When appropriate, percentage data were arcsine transformed prior to analysis by ANOVA. Linear regression techniques were used to examine the effect of level of zebra mussel meal addition on the AME of the diet. Egg mass and albumen height were used to calculate Haugh units (a measure of egg protein quality; Haugh 1937).

## Results

### Feed Composition

Proximate analysis is the partitioning of compounds in a feed into categories based on the chemical properties of the compounds (moisture, ash, crude protein, crude lipid, crude fiber, and digestible carbohydrates). It is based on the Weede analysis (Henneberg and Stohmann 1860). Table 1 contains the proximate analysis of the compounds that were available for pure mussel meal and the basal feed (crude protein, ash, and crude lipid). Zebra mussel meal was found to be low in fats and protein, and to have high ash content.

**Table 1:** Analyzed values of macro nutrient, amino acid, and gross energy content of basal feed and 100 % mussel meal

Nutrient (g kg^−1^)	Basal Feed	Mussel Meal
Nitrogen	28.1	5.2
Phosphorus	5.2	1.0
Calcium	34.8	344.9
Amino acids		
Aspartic acid	21.6	4.1
**Threonine**	9.1	1.6
Serine	9.3	1.8
Glutamic acid	37.3	3.7
Glycine	7.2	8.1
Alanine	7.5	1.6
**Valine**	9.7	2.0
**Methionine**	10.2	0.8
**Isoleucine**	8.9	1.5
**Leucine**	14.9	2.1
Tyrosine	5.1	4.2
**Phenylalanine**	10.0	2.1
**Histidine**	5.3	1.1
**Lysine**	12.2	1.8
**Arginine**	12.9	2.4
Proline	10.6	2.3
Gross energy MJ kg^−1^	15.3	0.8
Crude protein (N x 6.25)	175.6	32.5
Ash	110.0	928.0
Oil A (fats)	38.0	3.0

Amino acids in **bold** are the ‘essential’ amino acids that cannot be made by the body. Crude protein is calculated as N × 6.25 as N accounts for around 16 % of proteins (see Tituss 1961)

Analyses of macro nutrients, amino acid composition, and gross energy content of the two feeds revealed very low levels of energy present in the mussel meal (Table 1). Levels of calcium were around 10 times higher in the mussel meal than the basal feed, which is logical considering the high shell content. Zebra mussel meal was also high in glycine. Tables 2 and 3 compare amino acid composition and nutrient levels found in the zebra mussel meal used in the present study with the literature.

**Table 2:** Amino acid composition of the mussel meal (whole animal and shell) in this study (A) and comparative data from Secor et al. (1993), using zebra mussel soft tissues (B), and Jönsson (2009), using blue mussel (*Mytilus edulis*) soft tissues (C)

	Amino acids (g kg^−1^) Protein		
	A	B	C
Aspartic acid	99.0	75.1	73.1
Threonine	38.1	30.6	33.1
Serine	43.7	28.5	35.3
Glutamic acid	89.2	84.6	97.1
Glycine	197.5	6.2	40.4
Alanine	38.4	40.0	35.9
Valine	47.6	39.3	34.6
Methionine	20.4	16.6	17.7
Isoleucine	35.5	38.1	32.8
Leucine	50.0	56.7	50.2
Tyrosine	102.4	33.1	28.3
Phenylalanine	51.1	27.5	26.4
Histidine	25.7	12.5	14.5
Lysine	42.8	35.4	53.5
Arginine	57.3	49.7	53.2
Proline	56.5	28.4	27.3

**Table 3:** Comparison of N, P, and Ca levels found in zebra mussels in the literature. Whole mussels (body and shell) were used in the current study

Study	Flesh	Shell	Whole animal
Nitrogen (g kg^−1^)			
Current			5.2
Goedkoop et al. (2011)	100.9 ± 1.5		
Jurkiewicz-Karnkowska (2005)	120.41 ± 2.8	3.8 ± 0.5	
Stanczykowska (1984)	110–120.8	3.3	
Secor et al. (1993)	86.7–113		
Phosphorus g kg^−1^			
Current			1.0
Krolak and Zdanowski (2007)	6.6	0.45	
Stanczykowska (1984)	8.5–9.3	0.15	
Goedkoop et al. (2011)	9.3 ± 0.2		
Secor et al. (1993)	9.5–11.3	0.2–0.4	
Kuenzler (1961)	6–10	0.15	
Calcium (g kg^−1^)			
Current			344.9
Jurkiewicz-Karnkowska (2005)		370	
Krolak and Zdanowski (2007)	25.5	300.4	
Secor et al. (1993)		386–408	

### Production Performance

Common measures of production performance used in the industry for laying hens were calculated for each diet, and differences between treatments were assessed using one-way analysis of variance (Table 4). Voluntary feed intake by the birds did not change with diet. The amount of food it took to produce one gram of egg mass was not significantly different between diets (*P* = 0.84). Egg mass was lowest in the 15 % group; however, this difference was not significant. In all treatments, over 98 % of individuals laid an egg each day. The percentage of the excreta consisting of dry mass increased with percentage of mussel meal supplied in feed (*P* = 0.01). *Post* *hoc* tests (Bonferroni tests) showed that this difference lies between the control feed and both 7.5 % mussel meal and 15 % mussel meal. 15 % feed contained 4.5 % more dry mass than control feed.

**Table 4:** Production performance parameters for birds fed the three diets (± SE), and *P* values for one-way anovas are used to compare each characteristic by diet

Characteristics	Diet						F	d.f (between, within groups)	*P*
	Control	SE ±	7.5 %	SE ±	15.0 %	SE ±			
Change in bird bodyweight (kg)	−0.04^a^	0.02	−0.14^b^	0.03	−0.07^ab^	0.03	4.29	2,26	0.02*
Mean feed intake days 11–14 (g/bird/day)	105.13	3.33	104.33	7.19	105.47	5.34	0.01	2,26	0.99
Feed conversion (g food/g egg)	1.68	0.07	1.70	0.12	1.76	0.09	0.18	2,26	0.84
Excreta DM days 11–14 (g)	119.43	4.81	126.61	7.88	143.00	7.79	2.95	2,26	0.07
Excreta DM (%)	23.22^a^	0.96	26.02^b^	0.73	27.74^b^	1.25	5.29	2,26	0.01*
No. eggs/bird/day	0.99	0.01	0.99	0.01	0.98	0.01	0.26	2,26	0.77
Birds laying ≥1 egg/day (%)	98.57	0.95	98.57	0.95	98.41	1.05	0.01	2,26	0.99
Mean egg weight (g)	63.38	0.99	63.02	1.46	61.47	0.99	0.73	2,26	0.49
Mean deformation (µm)	22.00	1.26	22.50	0.87	20.22	0.79	1.36	2,26	0.28
Mean Haugh Units	108.09	1.53	106.65	1.27	111.12	1.81	2.15	2,26	0.14

Percentage values were Arcsine transformed prior to analyses. When *P* < 0.05, post hoc least significant difference tests were performed. Egg weight, deformation, and Haugh units were derived from eggs produced on days 11 and 12 of the trial. Letters ‘a’ and ‘b’ represent significant differences in the post hoc pairwise comparisons (Bonferroni tests)

Feed intakes are given on an ‘as fed’ wet weight basis

* Significant *P* value

A second parameter which differed significantly between diets was change in bird bodyweight. All treatments saw mean weight loss in the birds over the 14 day experimental period, however, the extent of this was significantly different between treatments (*P* < 0.05). *Post* *hoc* Bonferroni tests showed that the difference was significant between the control diet and 7.5 % mussel meal; those birds on the 7.5 % diet lost on average 0.1 kg more than those eating control feed.

### Apparent Metabolizable Energy

Due to the large difference in gross energy content of the basal and zebra mussel feeds (Table 1), AME was significantly affected by treatment *P* < 0.001; Table 6). The control feed differed significantly from both the 7.5 and 15 % mussel meal. AME was 1.29 MJ/kg less in 15 % mussel meal than in the control feed. Because of its low energy content, adding zebra mussel meal to the diet of a bird meant they had less available energy in their food. Linear regression analysis showed that zebra mussel content was negatively correlated with AME (*R* ^*2*^ = 0.528, *P* < 0.001). The intercept was 2.56 (SE ± 1.45), therefore the best estimate of metabolizable energy content of mussel meal was 2.56 MJ/kg, but this estimate is not significantly different from zero (*P* = 0.089).

### Amino Acid Digestibility

The proportion of each amino acid retained in the body compared to the amount ingested is important to assess the quality of a feed as a protein source. Digestibility varied between amino acids. However, between diets, there were no significant differences in digestibility (Table 5).

**Table 5:** Mean amino acid digestibility coefficient for each diet (±SE), and *P* values for one-way anovas, comparing digestibility by diet of each amino acid

Amino acid	Mean amino acid digestibility coefficient						F	d.f (between, within groups)	*P*
	Control	SE ±	7.5 %	SE ±	15 %	SE ±			
Aspartic acid	0.87	0.01	0.87	0.01	0.85	0.01	1.69	2,26	0.20
Threonine	0.86	0.01	0.86	0.01	0.85	0.01	1.02	2,26	0.38
Serine	0.87	0.28	0.87	0.27	0.85	0.28	0.72	2,26	0.50
Glutamic acid	0.91	0.01	0.90	0.01	0.88	0.01	3.19	2,26	0.06
Glycine	0.73	0.02	0.67	0.02	0.67	0.02	1.51	2,26	0.24
Alanine	0.74	0.01	0.75	0.03	0.75	0.01	0.04	2,26	0.96
Valine	0.83	0.01	0.82	0.02	0.81	0.01	0.52	2,26	0.60
Methionine	0.96	0.00	0.96	0.00	0.95	0.00	1.78	2,26	0.19
Isoleucine	0.86	0.01	0.86	0.01	0.84	0.01	1.67	2,26	0.21
Leucine	0.87	0.01	0.87	0.01	0.85	0.01	1.01	2, 26	0.38
Tyrosine	0.85	0.01	0.83	0.02	0.81	0.01	1.67	2,26	0.21
Phenylalanine	0.88	0.01	0.88	0.01	0.86	0.01	2.35	2,26	0.12
Histidine	0.87	0.01	0.84	0.03	0.85	0.01	0.65	2,26	0.53
Lysine	0.89	0.01	0.88	0.01	0.86	0.01	1.73	2,26	0.20
Arginine	0.92	0.01	0.91	0.01	0.90	0.01	1.96	2,26	0.16
Proline	0.88	0.01	0.87	0.02	0.86	0.01	0.27	2,26	0.76

### Retention of Other Nutrients

The amount of nitrogen, phosphorus, and calcium retained was calculated in the same way as for AME. There were no significant differences in retention for any of these three vital nutrients (Table 6) indicating that they were all well absorbed from the mussel meal. There was an increase in retention of nitrogen as more mussel meal was added to the diet, however, this was non-significant.

**Table 6:** Level of N, P, and Ca retained by diet (±SE), plus apparent metabolizable energy (AME), and *p* values for one-way anovas between treatments

Nutrient	Retention of nutrient (grams per kg feed intake)						F	d.f.	*P*
	Diet								
	Control	SE ±	7.5 %	SE ±	15.0 %	SE ±			
Nitrogen	13.44	0.21	11.76	0.78	12.02	0.33	3.13	2,26	0.06
Phosphorus	1.24	0.06	1.23	0.22	1.38	0.06	0.38	2,26	0.69
Calcium	19.43	1.04	25.37	3.19	27.48	4.04	2.00	2,26	0.16
AME	11.2^a^	0.113	10.5^b^	0.240	9.9^b^	0.097	14.63	2,26	<0.001*

AME is a measure of MJ retained per kilo of food consumed. Significant results are represented by an asterisk, and the letters a and b indicate a significant difference between groups (Bonferroni tests)

AME is given on an ‘as fed’ wet weight basis

## Discussion

### Proximate Composition and Nutrient/Amino Acid Levels of Basal and Mussel Feeds

Pure mussel meal was consistently shown to contain much lower levels of energy, protein, and nutrients than the basal control feed used. Gross energy was almost zero, and the other sources of energy, namely protein and fat, were also very low. However, the reduction in metabolizable energy with zebra mussel meal addition was entirely consistent with the amount of added mussel meal, and therefore addition of mussel meal itself did not have any deleterious effect on energy availability in the diet. However, at 344.9 g kg^−1^, calcium was present at levels ten times than those of the basal feed. This can be explained by the shell content of the zebra mussel meal, as all shells were included. The negligible energy levels may be down to the meal consisting almost entirely of shell material; which is very high in calcium but low in other nutrients. Calcium carbonate makes up 97 % of the eggshell of a chicken; therefore, calcium is essential for shell formation (Hunton 1995). A dietary level of around 4 % calcium is recommended, rising with the age of the bird (Cath et al. 2012). Limestone is the principally used calcium supplement; however, studies have shown oyster shell to have higher digestibility and retention, and to cause improvements in shell quality (Scott et al. 1971, Roberts 2004). This suggests that zebra mussel shell/whole animals could be used similarly, and indeed, the results of this study show that calcium from the mussel meal was well absorbed. The issue with using zebra mussels purely as a calcium supplement is that other sources are easily and cheaply available. Care would also need to be taken to avoid excessive calcium supplementation, as this could have a knock on effect on the digestibility of other minerals which could affect performance.

### Digestibility and Retention of Nutrients and Amino Acids

The extent to which the bird can retain, digest, and therefore utilise these nutrients is also vitally important. On the whole, this study showed that zebra mussel meal was a highly digestible and bioavailable material for poultry, and its use would not stop other nutrients from being absorbed. There were no significant differences for phosphorus, nitrogen, or calcium retention between the three diets. There was a numerical increase (not significant) in nitrogen retention as more mussel meal was added to the diet. This is logical as a lower protein intake might be expected to cause higher nitrogen retention to regain some of what was lost (Aletor et al. 2000). It is worth bearing in mind that excretion as well as retention of nutrients is important for a feed supplement, as excreted nutrients will have environmental impact.

Amino acid digestibility varied between individual amino acids, but was not significantly different between diets. Therefore, the amino acids present in mussel meal are perfectly digestible to chickens, but the very low levels of amino acids in the mussel meal compared with the same amount of control feed mean that the mussel meal used in this study would not be a suitable source of protein for chickens.

### Production Performance

We were able to monitor some essential production parameters, to ascertain whether introducing zebra mussel meal into a poultry diet could have any detrimental effects on production. Birds consuming all diets lost some weight during the study, with those on the 7.5 % diet having the highest loss. All other parameters including number of eggs per bird per day, feed conversion, and egg deformation (a measure of egg strength) did not vary with the addition of mussel meal. However, it should be noted that this was a very short study in terms of assessing production parameters. A similar pilot study using blue mussels found that the chickens actually preferred meal made from mussel flesh to conventional fodder (Lindahl et al. 2005). Excreta dry mass varied significantly between diets; increasing with mussel inclusion. This could be explained by the high calcium intake of birds on the mussel meal diets; increasing dropping dry matter content. Zebra mussel meal can therefore be assumed to have had no detrimental effects on production performance of laying hens.

### Application of Mussels for Chicken Feed

Mussel biomass may become available as a waste product from a number of sources. The increasing interest in using bivalves as a tool for nutrient removal and clarification in eutrophic waters (Lindahl et al. 2005; Stybel et al. 2009; McLaughlan and Aldridge 2013) can generate tonnes of material. Proposed uses of the removed mussels include human food, fertilizer, animal feed, and biogas (Stybel et al. 2009). The first three uses have been successfully tried with the marine mussel *Mytilus edulis*. These mussels (flesh only) were used for a comprehensive pilot scheme where they were fed to both laying and broiler chickens, with no ill effects on production parameters reported (Jönssons 2009). In Jönsson’s study, the mussel flesh was found to be high in essential amino acids, such as methionine, and mussel meal produced darker orange egg yolks and in some cases higher plumage conditions in the birds than a control feed. Jönsson’s study involved only the flesh of the mussels. Separating flesh from shell could be tried with zebra mussels, and indeed, this is being trialled in another pilot study in Sweden with some success (O. Lindahl, pers. comm). According to Secor et al. (1993), zebra mussel flesh contains amounts of amino acids almost identical to those of *Mytilus edulis* reported by Jönsson (2009). There is no reason, therefore, why they cannot provide a good source of protein. The other option would be to use the whole mussel, and utilise the potential of the zebra mussels as a calcium supplement for chickens.

Other relevant issues include adverse effects on the taste of the eggs and accumulation of toxins in zebra mussel flesh. Although no scientific trial of egg taste was conducted here, eggs from the study were consumed by the author on several occasions and no undesirable flavors were noted. Lindahl et al. (2005) found the same with eggs from hens that had consumed blue mussel flesh. In terms of toxins, some authors have concluded that zebra mussels contain minimal amounts of harmful substances (Doherty et al. 1993; Kreis et al. 1994), but this would depend on the quality of water they had filtered and would be considered on a case by case basis.

In conclusion, whole zebra mussels have been shown to be an innocuous addition to egg-laying poultry feed, providing high levels of the essential nutrient calcium. A zebra mussel-supplemented diet showed no detrimental effects on production performance, and was palatable to the birds. The low protein content of whole animals means that they must either be separated from their shells or used whole as more of a calcium supplement. The success of using zebra mussels as a chicken feed supplement will therefore depend on economic factors, such as the cost of processing and the price (if any) that could be demanded per tonne of zebra mussel meal. Such economic calculations must be balanced against the costs associated with alternative disposal routes. With one UK water company having to dispose of ca. 2000 tonnes of zebra mussel biomass per year to landfill (M. Chipps, Thames Water, pers. comm.) with a landfill fee alone of approximately £150,000 (based on 2013 prices), the economic and sustainability benefits are likely to favor creative end-use of mussel biomass.
